# Isolated Central Nervous System Metastasis From Neuroendocrine Carcinoma of the Cervix Without Pulmonary Metastasis

**DOI:** 10.7759/cureus.10728

**Published:** 2020-09-30

**Authors:** Fabiola Valenzuela, Sohum Desai

**Affiliations:** 1 Department of Surgery, University of Texas Rio Grande Valley School of Medicine, Edinburg, USA; 2 Department of Neurological Surgery, BHS Physicians Network, Harlingen, USA

**Keywords:** neuroendocrine carcinoma of cervix, isolated brain metastasis, craniospinal radiation

## Abstract

Neuroendocrine carcinoma of the cervix (NECC) accounts for 2% of all cervical cancers. Brain metastasis is rare, with few cases described in the literature, and is usually associated with preceding pulmonary metastasis. We describe an additional case of isolated brain metastasis without pulmonary metastasis from NECC and reflect on unique management.

A 37-year-old woman with a history of NECC presented with severe headache post-total hysterectomy with pelvic lymph node dissection. The computed tomography (CT) scan demonstrated obstructive hydrocephalus with several intra-axial lesions located in the pineal region, left cerebellar hemisphere, and left frontal operculum. A right frontal ventriculostomy was initially placed to relieve the hydrocephalus. CSF was sent for cytology but was unrevealing. Due to the degree of brainstem compression and the need to obtain a pathologic diagnosis, a posterior fossa craniotomy for the removal of the lesion was performed. Histopathology demonstrated small blue cell tumors positive for neuroendocrine markers consistent with neuroendocrine carcinoma of the cervix. Resection of additional metastasis was not recommended. An endoscopic third ventriculostomy (ETV) was then performed in order to remove the ventriculostomy with success. The patient was then referred to radiation oncology and received whole-brain radiotherapy (WBRT) for a total of 30 Grays (3000 cGy) over 10 fractions. Interval imaging demonstrated complete resolution of the pineal and left frontal lesions. The patient was symptom-free for approximately three months. She then presented with paraplegia consistent with follow-up imaging of her neuraxis, demonstrating drop metastasis in her cervical, thoracic, and lumbar spine. Spinal radiation was given with partial recovery in upper extremity function, however, lower extremity function did not recover. The patient was then transferred to palliative care.

There are no guidelines on NECC brain metastasis management. Brain metastasis is associated with reduced longevity. NECC has a propensity for early dissemination and treatment failure. ETV is preferred over ventriculoperitoneal shunting in cases with obstructive hydrocephalus, as it may reduce the risk of tumor seeding. Retrospectively, our patient may have benefitted from upfront craniospinal radiation.

## Introduction

Neuroendocrine carcinomas are derived from neuroendocrine cells, which originate from the embryonic neuroectoderm. Neuroendocrine carcinomas are divided into well-differentiated (typical and atypical carcinoid) and poorly differentiated (small cell and large cell), with small cell being the most common subtype [1]. Neuroendocrine carcinomas of the cervix (NECC) are rare, accounting for 2% of all cervical cancers [2]. The average age is 45 years, and survival averages 6.4 months after initial diagnosis for those with advanced disease [3-4]. Given the rarity of NECC, guidelines appear to be based on large case series [1,5]. Metastatic NECC to the central nervous system (CNS) is rare and difficult to manage, with few cases of brain metastasis specifically having been reported [6]. Here, we describe an illustrative case of isolated brain metastasis in NECC and points to consider for the management of this entity.

## Case presentation

**Figure 1:**
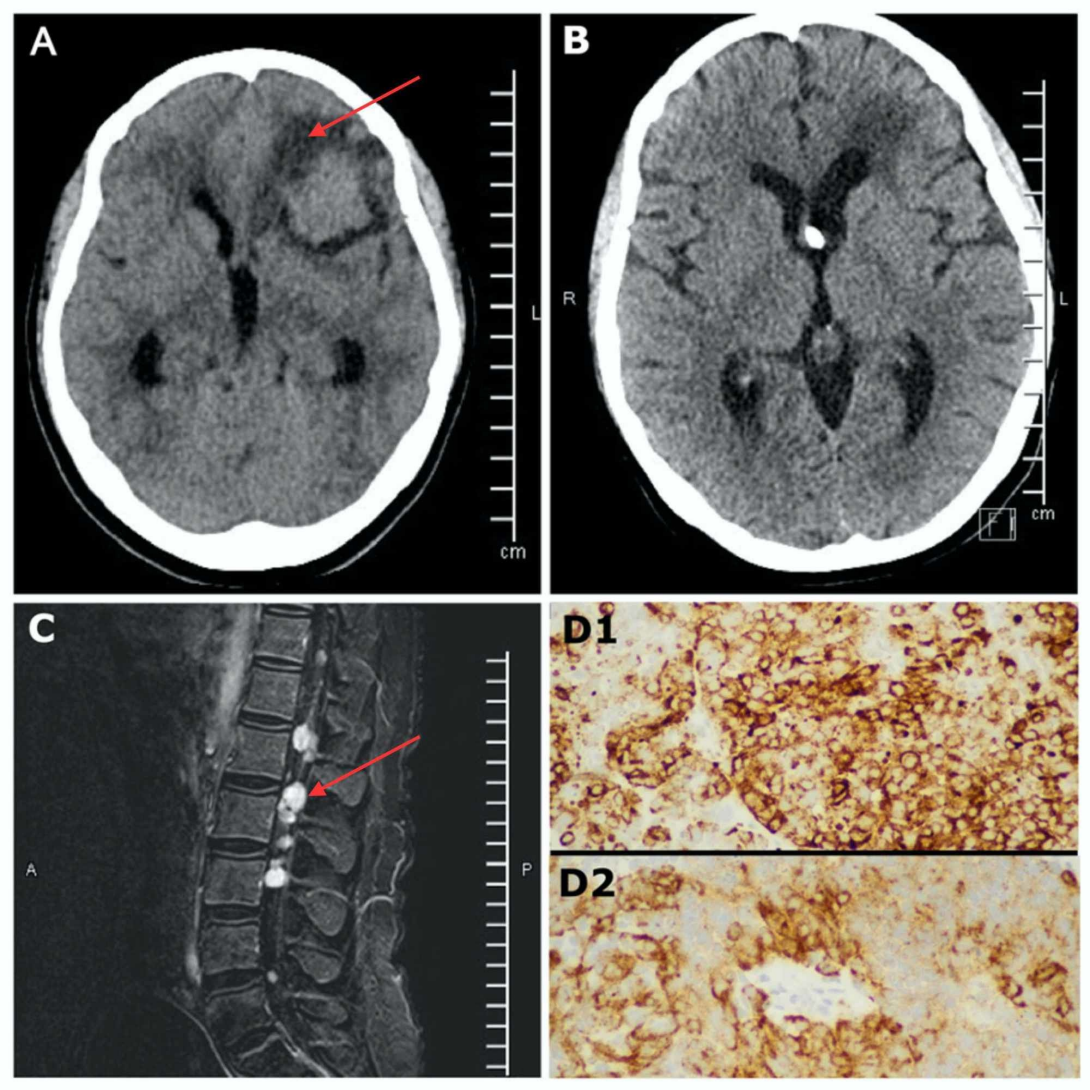
A) CT head without contrast shows a left frontal mass associated with vasogenic edema (red arrow). There is obstructive hydrocephalus from a posterior fossa and pineal lesion not pictured. B) CT head without contrast obtained approximately one month after the initial biopsy, endoscopic third ventriculostomy, and cranial radiation. There is resolution of hydrocephalus and the left frontal mass has disappeared. There is a small amount of encephalomalacia where the mass was located. The right frontal catheter belongs to a reservoir inserted for the ETV. C) Sagittal T1 post-contrast MRI demonstrating an enhancing intradural extra-medullary lesion (red arrow) consistent with drop metastasis. D) Uniform tumor cells with salt and pepper nuclear chromatin: D1 shows a photomicrograph of cells positive for neuroendocrine chromogranin marker stain at 40X from biopsy, D2 shows a photomicrograph of cells positive for a neuroendocrine synaptophysin marker stain at 40X from biopsy. CT: computed tomography; ETV: endoscopic third ventriculostomy; MRI: magnetic resonance imaging

A 37-year-old female with a known history of neuroendocrine carcinoma of the cervix (NECC) for the past 11 months who had undergone a partial hysterectomy presented to the neurosurgical service with a severe headache unresponsive to medical treatment after recently undergoing a follow-up completion hysterectomy with pelvic lymph node dissection. Figure 1 presents imaging and pathology findings. A CT head without contrast demonstrated florid obstructive hydrocephalus and several intra-axial lesions occupying the pineal region, left frontal lobe, and posterior fossa (Figure 1A). A right frontal ventriculostomy was placed to temporize the hydrocephalus. Due to the degree of brainstem compression and the need to obtain a pathologic diagnosis, it was decided to proceed with a posterior fossa craniotomy for the removal of a single lesion. The frozen pathology yielded a small blue cell tumor with positive neuroendocrine markers cluster of differentiation 56 (CD56), chromogranin, and hematoxylin and eosin (H&E) stain consistent with a prior diagnosis of neuroendocrine carcinoma of the cervix (Figure 1D). The remaining lesions were left alone given the biologic responsiveness of neuroendocrine carcinoma to radiation therapy. An endoscopic third ventriculostomy (ETV) was then performed to remove the ventricular drain. The patient was then discharged from the hospital. She then went on to receive whole-brain radiotherapy (WBRT) for a total of 30 Grays (3000 cGy) over 10 fractions on an outpatient basis. A follow-up CT head without contrast was obtained (Figure 1B) that demonstrated the resolution of the pineal and left frontal lesions. The patient returned for follow-up ambulatory without any major concern. Subsequently, she presented to the oncologist with quadriparesis three months later. Magnetic resonance imaging (MRI) was obtained, which demonstrated drop metastasis in the cervical, thoracic, and lumbar area (Figure 1C) most likely related to biopsy, explaining the quadriparesis presentation. She had further spinal radiation of 30 Grays (3000 cGy) over 10 fractions. Her neurologic function improved to antigravity in the upper extremities, however, she remained paraplegic. Palliative care and hospice followed.

## Discussion

Brain metastasis is rare in NECC. There are no guidelines on how to manage brain metastasis when it reaches this stage. Per the Society of Gynecologic Oncology (SGO), treatment, especially chemotherapy, is generally translated from cervical cancer and small cell lung cancer (SCLC) management, given the histological and clinical similarities [1]. For SCLC, prophylactic craniospinal radiation has resulted in a better prognosis and increased survival rates [7]. However, prophylactic craniospinal radiation is not routinely given for early-stage NECC.

Our patient had a survival of 13 months from diagnosis. Given that her neurologic status declined due to spinal metastasis after the complete resolution of her cranial lesions, our patient may have benefited from prophylactic craniospinal radiation upfront and maintained her ambulatory status longer. This is apparently in contrast with current guidelines that do not recommend prophylactic craniospinal radiation in patients with early NECC.

While brain metastases are typically observed concurrently with lung metastases [8], our case bears some resemblance to others in the literature that describe isolated brain metastasis [9-11]. Weed et al. reported approximately 25% development of brain metastasis in patients with early-stage small cell cervical cancer, and proposed the use of prophylactic cranial radiation for better survival outcomes [12]. Recent literature has suggested reduced longevity in patients with brain metastasis from cervical cancer independent of whether metastasis is isolated to the brain, with a median survival of 7.63 months after initial diagnosis [13]. Viswanathan et al. discourage the use of craniospinal radiation prophylactically in the early stage [8].

The development of guidelines for the management of NECC and metastasis are needed to better guide treatment. Further research looking at the management and outcomes of NECC brain metastasis and the use of craniospinal radiation is needed. Research studying other management, including stereotactic radiosurgery and multimodal approaches, would be beneficial. There are potential opportunities for investigations into determining what mutations are responsible in NECC to allow for CNS penetration through the blood-brain barrier. It would also be of interest to study the incidence of microscopic CNS disease.

It is important to acknowledge some limitations. The use of prophylactic craniospinal radiation is often at the discretion of the treating physician or radiation oncologist given that there are no established guidelines for the management of NECC. There is limited research on survival rates with the use of prophylactic craniospinal radiation. Whether the type of NECC plays a role in increasing longevity with the use of prophylactic craniospinal radiation is not fully understood.

## Conclusions

Brain metastasis of NECC is associated with reduced longevity and has a propensity for early dissemination and treatment failure. Our patient with isolated CNS metastasis from NECC may have had longer ambulatory status with craniospinal radiation. In our patient with obstructive hydrocephalus, ETV was preferred over ventriculoperitoneal shunting to reduce the risk of tumor seeding. The development of guidelines on NECC brain metastasis management is needed.
